# Food environment change on wild food consumption in rural Tanzania

**DOI:** 10.1007/s12571-024-01469-6

**Published:** 2024-08-20

**Authors:** Rasmus Skov Olesen, Bronwen Powell, Charles Joseph Kilawe, Laura Vang Rasmussen

**Affiliations:** 1 University of Copenhagen, Copenhagen, Denmark; 2 The Pennsylvania State University, University Park, USA; 3 Sokoine University of Agriculture, Morogoro, Tanzania

**Keywords:** Wild foods, Diet change, Landscape change, Food environments, Tanzania, Longitudinal study

## Abstract

In this longitudinal study we explore how changes in food environments have shaped the acquisition and consumption of wild foods among people living near forests. Our study conceptually improves food environment frameworks by including evidence on changes in wild food consumption. We used data collected in both the dry and rainy seasons in 2009 and 2021/2022 in four villages in the East Usambara Mountains, Tanzania. Across data collections, we conducted qualitative interviews, focus groups and repeated household surveys, including questions on dietary intake, food sources, agricultural practices, and use of wild resources. We found that the proportion of people who collected wild foods within the past seven days had declined from 90 to 61% in the dry season and from 99 to 72% in the wet season. The main reasons were 1) decreased *availability* caused by, for example, loss of biodiversity, 2) lack of *access* due to government forest regulations, and 3) increased *desirability* towards marked-based foods. Our results show how changes in both availability, access and desirability of wild foods have shifted dietary choices from wild foods towards cultivated and purchased foods. Also, we see less widespread consumption of sentinel food groups such as dark green leafy vegetables. Our results highlight the need for an additional dimension in existing food environment frameworks: “Legal access to wild resources” that would cover access to wild foods. This dimension is important as loss of legal access and declining consumption can have negative dietary implications, since the most commonly consumed wild foods, such as leafy vegetables, are nutritionally important.

## Introduction

There is a critical need to identify solutions to current and future challenges of the world’s food system. One of the main challenges is that nearly one in three people in the world (2.37 billion) suffer from malnutrition (FAO et al., 2022; HLPE, 2020). Global food systems have undergone dramatic changes in recent decades, including homogenization of global diets (Khoury et al., 2014) and substantial increases in agricultural productivity and expansion of crop lands over the past 50 years (FAO, 2021). The global agricultural system currently overproduces grains, fats, and sugars (Kc et al. 2018), yet fails to provide enough fruits and vegetables to meet global requirements (Siegel et al., 2014). In parallel with the insufficient production of fruits and vegetables, global diets have shifted away from reliance on traditional foods towards a higher dependence on purchased and often highly processed foods, and higher intake of fat, salt and sugar (Popkin et al., 2020). This is worrying from a food and nutrition security perspective, as far more people suffer from micronutrient deficiencies than undernourishment (Bailey et al., 2015; Kennedy et al., 2003).

To conceptually examine the implications of these dietary trends within local settings, a number of studies have applied the food environment framework (Turner et al., 2020)*.* The food environment framework is an analytical approach used to examine structural and environmental factors that shape dietary choices and, more recently, dietary transitions. The food environment includes aspects of the food systems that people navigate and interact with when making decisions about their food consumption, such as availability, affordability, convenience, and desirability (FAO, 2016; Herforth & Ahmed, 2015; Swinburn et al., 2015; Turner et al., 2018). A food environment framework has been proposed as an important tool to understand the links between agricultural systems and dietary transitions (Downs et al., 2020). However, with the exception of Downs et al. (2020), wild foods, which are often nutrient-dense (Blaney et al., 2009; Fungo et al., 2016; Golden et al., 2011; Vinceti et al., 2013), have been generally overlooked by food environment frameworks to date.

This paper explores two questions: 1) How do changes in the food environment affect wild food acquisition and consumption? And 2) how can a case study of changes in wild food acquisition and consumption help to conceptually improve the food environment framework, so that it is better suited for rural settings? Answering these two questions has important implications for strategies to achieve food and nutrition security, particularly in rural regions of low- and middle-income countries (LMICs), which previously exhibited strong connections between people’s diets and landscapes, and where malnutrition (especially micronutrient deficiency) remains highly prevalent (FAO et al., 2022).

### Wild foods in transitioning agricultural systems

The mainstream agricultural sector’s focus on increasing outputs and “closing the yield gap” has led to intensification of agriculture in LMICs. As a consequence, many rural areas in LMICs have experienced increased commodity production and improved market access, which in some settings has provided better income opportunities and higher dietary diversity (Jones, 2017; Meemken & Bellemare, 2020; Nandi et al., 2021; Sibhatu et al., 2015). However, agricultural intensification and commercialization has in multiple cases also failed to support food and nutrition security (Carletto et al., 2017; Dewey, 1989; Ickowitz et al., 2019; Rasmussen et al., 2018). For example, increased agricultural commodification has been associated with food transitions away from traditional food systems, which has led to lower dietary quality among rural populations – especially when looking at people’s dietary diversity and their intake of important micronutrients (Akombi et al., 2017; Chegere & Stage, 2020). A recent longitudinal study from the Amazon found that communities having undergone expansion of market-oriented agriculture and deforestation had lower average household dietary diversity, greater reliance on purchased food and less wild food consumption (Blundo-Canto et al., 2020). Another study from Guatemala compared one community that had adopted palm oil production to one that had not, and reported uncertain food supply and, less hunting, fishing, and wild plant collection in the palm oil community. Both communities reported decreases in the consumption of nutritious perishable foods and cited declining availability as the primary reason for a decrease in consumption of vegetables, fruits, herbs, fish, and game meat (Hervas, 2020; Hervas & Isakson, 2020).

Focusing on the consequences of lower wild food consumption is key, as it is well-established that wild foods (especially wild plant foods) are essential supplements to rural diets in LMICs (Asprilla-Perea & Diaz-Puente, 2019; Campbell et al., 2021; Golden et al., 2011; Guyu & Muluneh, 2015). In the Food and Agriculture Organization of the United Nations’ (FAO) report ‘The State of Biodiversity for Food and Agriculture’, 15 countries reported regular use of wild foods by the majority of the population and 26 reported regular use of wild foods by a subsection of the population (Pilling & Bélanger, 2019). Some communities reported using over 120 wild food species (Bharucha & Pretty, 2010), and a study based on data from 7845 households in 24 LMICs found that 77% of the households were engaged in wild food collection (Hickey et al., 2016). A study of the importance of wild foods in forest-adjacent communities in the tropics showed large variation in the use of wild foods between communities in the same country (contributing on average 14% of fruits and vegetables consumed, but varying from 0–96%) (Rowland et al., 2017). Although wild foods rarely represent a major source of calories, they often contain high levels of essential micronutrients that can be difficult and expensive to acquire elsewhere (Blaney et al., 2009; Fa et al., 2015; Ickowitz et al., 2016; Powell et al., 2013). A study among rural women in Cameroon found that wild foods from the forest made significant contributions to local intakes of vitamin A (93%), sodium (100%), iron (85%), zinc (88%), and calcium (89%) (Fungo et al., 2016). And a recent study from central Amazonia found that wild meat consumption was associated with higher hemoglobin levels among rural children and that the loss of these wild food sources may lead to a 10% increased prevalence of anemia among extremely poor children (Carignano Torres et al., 2022).

Some studies report greater reliance on wild foods in times of crisis, during seasonal and transient food insecurity (Fungo et al., 2016; Somnasang & Moreno-Black, 2000; Sunderland, 2011). Despite some studies questioning the safety-net argument (Paumgarten et al., 2018; Wunder et al., 2014), the hidden harvest of wild foods (Scoones et al., 1992) has experienced renewed attention in the light of the COVID-19 crisis and food insecurity levels being on the rise in many countries (Bhushi, 2021; Hall, 2021).

In parallel with dietary transitions, wild foods are disappearing from diets in many communities (Borelli et al., 2020). Greater market integration and agricultural change has been associated with lower use of wild foods in a number of different small-scale subsistence communities (Reyes-García et al., 2019; Schlegel & Guthrie, 1973) and climatic changes are seen as a threat to many traditional wild foods (Bezner Kerr et al., 2022). A study, using data from 751 households across four African countries, found that higher tree and natural grassland cover significantly predicted whether a household would report collecting wild foods (Cooper et al., 2018). Another study identified a number of threats to wild food plant use, including changes in land use, climatic changes, overexploitation, urbanization, and loss of traditional knowledge and practices associated with their use (Borelli et al., 2020).

### The food-environment framework and the limited attention to wild foods

Most food environment research to date has predominantly focused on purchased foods in built food environments (Hua et al., 2014; Pehlke et al., 2016; Phulkerd et al., 2017; Rathi et al., 2017), while wild foods have been overlooked. For example, Turner et al. (2020) found only one publication that paid specific attention to wild foods in their scoping review of 70 food environment publications. Also, Constantinides’ (2021) assessment of 15 different research projects on food environments in LMICs showed that only one project attended to consumption and acquisition of wild foods.

The need to incorporate wild foods in food environment frameworks has been pointed to by a number of scholars (Herforth & Ahmed, 2015; Powell et al., 2015). Downs et al. (2020) recently proposed that nutrition transitions around the world can be mapped onto what they call ‘food environment transitions’. They identify four different types of food systems: Wild, cultivated, informal markets, and formal markets. They propose that - as countries and communities pass through the phases of a nutrition transition - they shift from relying on wild food environments, to cultivated, to informal and then to formal market food environments (Downs et al., 2020; Popkin et al., 2020).

Yet, with almost no food environment research including wild foods, the empirical evidence for making general conceptualizations of the early phases in these transitions is limited. In this paper, we present our results from a longitudinal study spanning over more than ten years. We apply a food environment framework to understand the changes in consumption of wild foods in rural forest-adjacent communities in Tanzania where widespread use of wild foods has been documented (Fleuret, 1979; Keding et al., 2007; Powell et al., 2013). Herein, wild food is defined as ‘*uncultivated species (plant or animal) that live and grow spontaneously, instinctively and locally in nature, with no intervention or control by people’.*

**Fig. 1:**
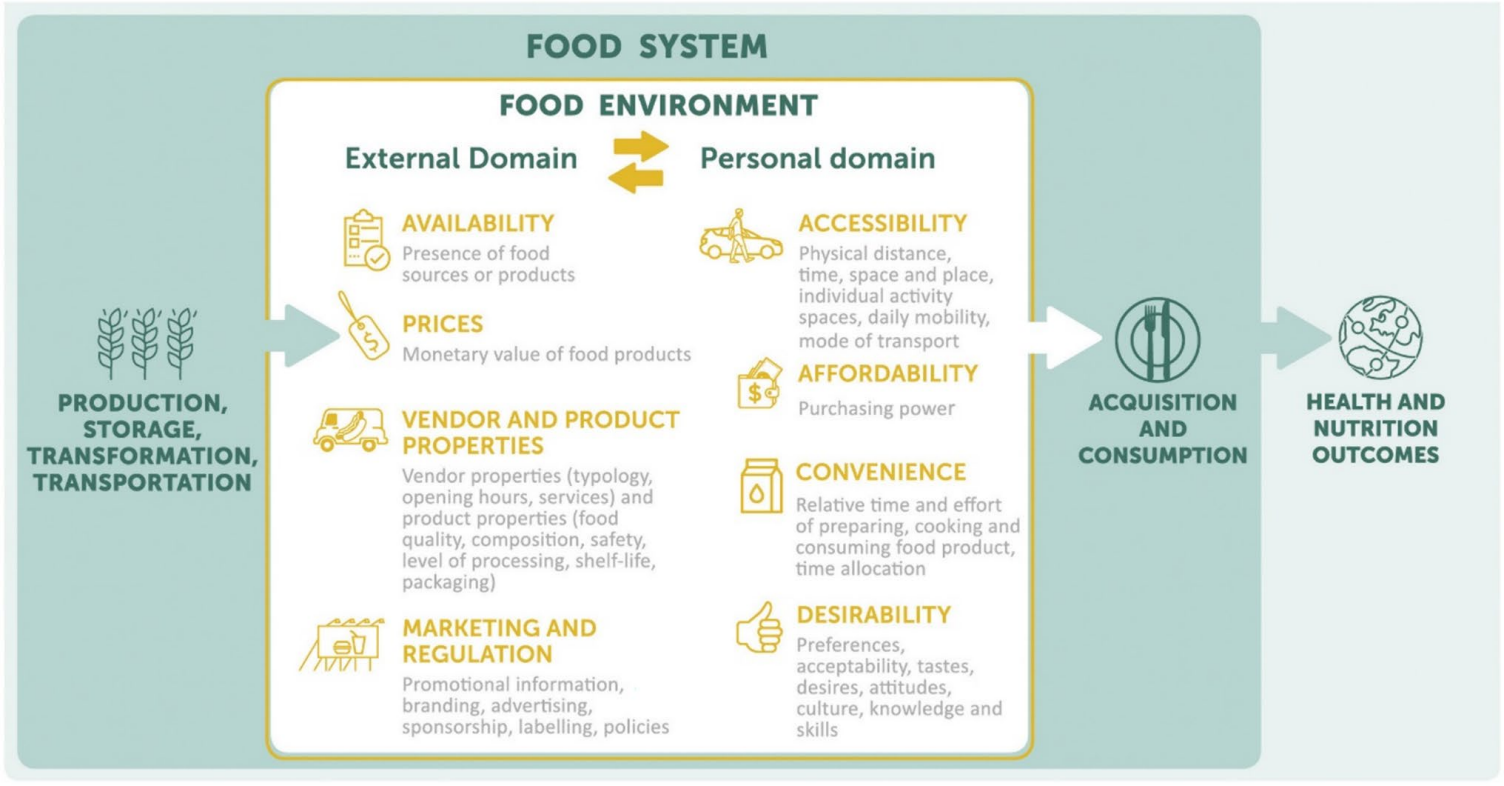
The conceptual food environment framework as proposed by Turner et al. (2018). License provided by Elsevier

We draw on Turner et al.'s (2018) version of the food environment framework, designed for research in LMICs, as we find the conceptual categories most nuanced and well-suited for our coding strategy. The framework includes an external and a personal domain (Fig. 1). The external domain refers to the factors that either provide or constrain opportunities for food acquisition in a given system – and these do not vary across individuals within the ‘same’ system. The external domain includes four dimensions: 1) Availability, 2) prices, 3) vendor and product properties, and 4) marketing and regulation. The personal domain refers to the features that determine how individuals make decisions within their food environment, and it includes four dimensions: 1) Accessibility, 2) affordability, 3) convenience, and 4) desirability. In addition to these, we propose the need for an additional dimension, necessary to adequately understand choices around the consumption of wild foods: *Legal access to wild resources*.

## Materials and methods

### Study site

Tanzania provides a unique case for examining the role of wild foods in food environments as wild foods provide important dietary supplements for many rural communities (Ceppi & Nielsen, 2014; Kaya & Lyana, 2014; Murray et al., 2001; Ruffo et al., 2002). Moreover, finding solutions to improve food and nutrition security continues to be a grand challenge in the country. Although Tanzania has experienced over 20 years of sustained economic growth, culminating in its transition from low-income to lower middle-income status in 2020, the country still exhibits high rates of hunger and malnutrition (FAO et al., 2022; The World Bank, 2022). Approximately 3 million children under 5 years (31.8%) were stunted in 2018 (MoHCDGEC et al. 2018), and 25.8% of the total population suffers from severe food insecurity (FAO et al., 2022).

Early work by Fleuret (1979) demonstrated the central role of wild foods in the diets of rural communities in Tanzania and more lately a study among 359 households from four different districts in Central and Northern Tanzania found that 93% of respondents perceived wild foods to be important contributors to diets in times of food shortage (Weinberger & Swai, 2006). Another recent study from Northern Tanzania among children aged 5 to 14 years found that 71.4% of the boys were involved in foraging activities (Pollom et al., 2020).

The country hosts high levels of biodiversity and around 30% of the population live within 5 km of a forest (Newton et al., 2020). A study has shown that people living with more forest cover and a higher number of forest patches in their surroundings are more likely to consume fruits as compared to people with less forest in their surroundings (Rasmussen et al., 2019). Moreover, Hall et al. (2022) have demonstrated a causal relationship between forest loss and reduced fruit and vegetable consumption (of 14 g per person per day) in Tanzania. Meanwhile, the country’s forests and wild resources are under increased pressure from agricultural expansion and deforestation (Burgess et al., 2002; Capitani et al., 2019).

### Study design and data collection

This paper presents the results of a longitudinal, mixed methods study that took place in two rounds of data collection (2009 and 2021/2022). Each data collection round included household surveys with a focus on dietary intake information as well as focus group discussions and interviews (Table 1). During each round, data were collected in both the rainy and dry season to account for seasonal differences in food intake.

**Table 1:** Overview of data collection and field methods

**Rounds of data collection**	**Principal investigator**	**Focus groups**	**Semi-structured interviews**	**Ecological data**	**Household survey and diet data**
2009 (March–May) Wet season	Bronwen Powell	8 focus groups (2 per village × 4 villages) with village leaders/local experts. Food list, local calendars, participatory ranking (N = ~ 80)	None	Voucher collection and identification by expert botanist	Repeated 24 h dietary recalls (N = 184) mothers and children in 4 villages
2009 (Sept-Oct) Dry season	Bronwen Powell	5 focus groups with women in 5 villages, on drivers and constraints to diet quality and wild food use (N = 34) (completed in 2012)	Key informant interviews over multiple days in 15 households in 6 villages	Voucher collection and identification by expert botanist	24 h dietary diversity and source of food (N = 129) mothers and children in 3 villages
2021 (Oct) Dry season	Rasmus Skov Olesen	4 focus groups with village leaders. (N = 21). Timeline exercises	8 semi-structured interviews with selected respondents and key-informants.	4 transect walks.	Household surveys, including repeated 24 h dietary recalls (N = 239).
2022 (March) Wet season	Rasmus Skov Olesen	4 focus groups with selected respondents. (N = 19). Ranking exercises on wild foods.	None	4 transect walks.	Household surveys, including repeated 24 h dietary recalls (N = 212)
		4 focus groups with village leaders. (N = 23). Timeline exercises.			
**Main objectives:**		Changes in food sources (cultivated, purchased/gifts and wild collection) Changes in external and personal food domains Description and evaluation of different wild foods Changes in land use, forest access, legal access to wild resources and agricultural practices.	Changes in food habits (food sources, preparation, cooking)	Identification and description of forest access, forest types, legal access to wild resources, wild foods and agricultural practices	Basic household information, income activities, use of forest resources, agricultural practices, dietary data.
**Food Environment dimensions**		Availability, marketing and regulation, prices, access, affordability, convenience and desirability	Access, affordability, convenience and desirability	Availability, marketing and regulation, access and convenience	Availability, access, affordability

#### Focus groups

In 2022, we conducted four focus group discussions that included ranking exercises with women who participated in a similar focus group in 2009. The aim was to evaluate the participants’ acquisition and consumption of wild foods over time. Fifteen participants from the original 2009 focus groups were included in the focus group interviews in 2022. It was, however, not possible to find all participants from 2009 as some had moved to other areas or were not available. We began the focus group sessions by cross-checking participant names and showing photos of the principal researcher from 2009 to confirm that they had also participated in 2009.

**Fig. 2:**
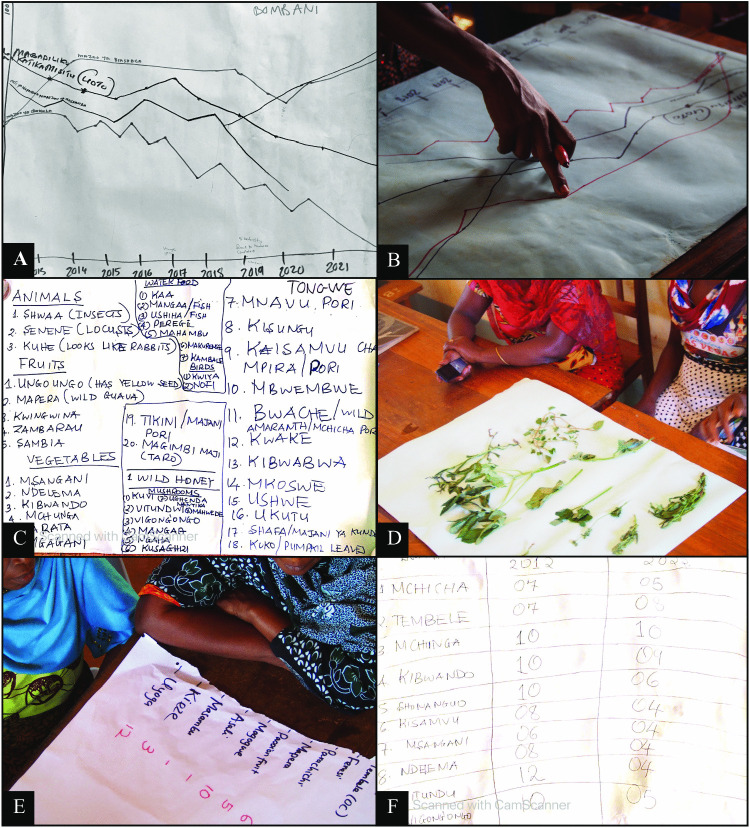
Focus group discussions. The focus group discussions included timeline exercises (**A** and **B**) and ranking exercises (**C**–**F**)

Key-questions on reasons behind changes in acquisition and consumption of wild foods from the focus group protocol in 2009 were replicated in the protocol in 2022 to assess changes over time. The participants were asked to name the wild foods available in the area over the entire year (Fig. 2C). Based on the list, they were asked to choose the ten most important wild foods (Fig. 2E). Participants were then given a handful of dried beans each and asked to estimate how many days in a given week they would consume each of the selected wild foods in 2009 vs 2022 by adding the corresponding number of dried beans to the board. The total number of beans added to the board by all participants was recorded in the matrix table (Fig. 2F). The matrix was used to discuss reasons behind changes in wild food acquisition and consumption, not to provide exact estimates of changes in wild food consumption. The focus group aimed to bring out different opinions rather than reach consensus among participants. The focus group protocol included open questions on the drivers and barriers to wild food acquisition and consumption. Also, when specific dimensions of the food environment framework were not mentioned, we asked additional questions to ensure that we covered all dimensions.

Eight of the focus groups (four in 2021 and four in 2022) included timeline exercises to capture changes in forest use, agricultural practices, market access, and food consumption (Fig. 2A). Here, participants were asked to draw trend lines for forest cover, food crop production, cash crop production, and general food availability (Fig. 2B). The aim was to examine larger contextual changes surrounding wild food consumption, as perceived by the participants. We did not aim to extract precise information regarding exact points in time. The participants were encouraged to reach consensus on the drawn timelines. However, we welcomed an open discussion with different opinions during the entire session.

#### Surveys

**Fig. 3:**
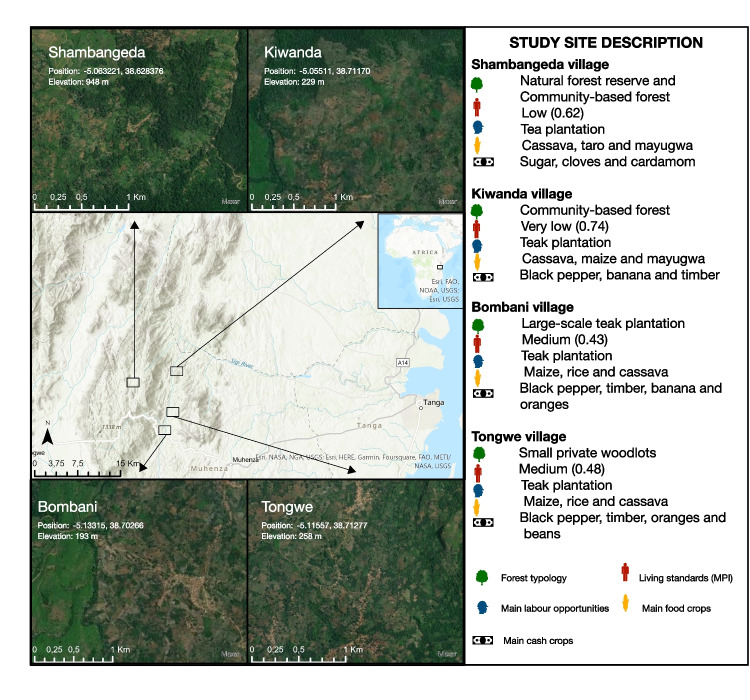
Map of study sites. East Usambara Mountains are located in Muheza district, Tanga Region in Northeastern Tanzania (-5° 04′ 58.80" S 38° 40′ 1.20" E). The villages differed both in terms of forest management, types of crops and average living standards (see Sect. 2.3.2. for further explanation on calculation of Multidimensional Poverty Index (MPI) living standard

In 2009, six villages were selected along gradients of distance to market (the town of Muheza) and distance to protected area, as well as elevation levels. In 2021/2022, four villages were selected from the original six based on differences in forest characteristics to capture different types of access to wild resources (Fig. 3). Two of the original villages were not revisited due to limited resources. The data from these two villages collected in 2009 were excluded from the analysis.

The household surveys were carried out among women with children under 5 years. The household surveys from 2009 and 2021/2022 included information on basic household characteristics (e.g., age, educational level, household composition), agricultural practices (e.g., land size, livestock ownership, crop production, homegardens, trees on farms), food consumption (e.g., types of food, quantities and sources), use of forests (e.g., trips to forest, collection of non-timber forest products (NTFPs)) and wild food consumption (e.g., types of wild foods and quantities). We replicated the survey form across data collection rounds to ensure comparability.

During the first study in 2009, 45 respondents were selected in each of the six villages using a random sample frame and a list of children under 5 provided by village leaders. The same respondents were visited during the wet (N = 184) and dry season (N = 129), except that some respondents were not available during the dry season and one of the villages could not be sampled due to insufficient resources. In 2021/2022, 60 respondents were selected from each of four of the original six villages through stratified random sampling, weighted by the proportion of residents in each hamlet (sub-village) and chosen from a list of women with children under 5 years provided by the village leaders.

### Data analysis

#### Qualitative data

Focus group summaries and interview transcripts were organized, coded and analyzed using Nvivo (version 12). The program has the advantage that it allows for analysis across large amounts of qualitative data and different types of empirical material (Jackson & Bazeley, 2019). We used directed (deductive) coding based on all dimensions of the food environment (availability, prices, vendor and product properties, marketing and regulation, access, affordability, convenience and desirability) (Saldaña, 2021). However, we note that our study methods did not illicit information on changes in the category ‘vendor and product properties’. Our results on change in forest access and food prices are based on local communities’ expertise and perceptions rather than ecological assessments or market surveys of prices.

To assess changes over time, we coded all qualitative data according to year (2009, 2021 and 2022) and season (wet and dry). This enabled us to detect qualitative differences within each of the food environment dimensions (e.g., description of food preferences) across the years.

#### Quantitative data

**Fig. 4:**
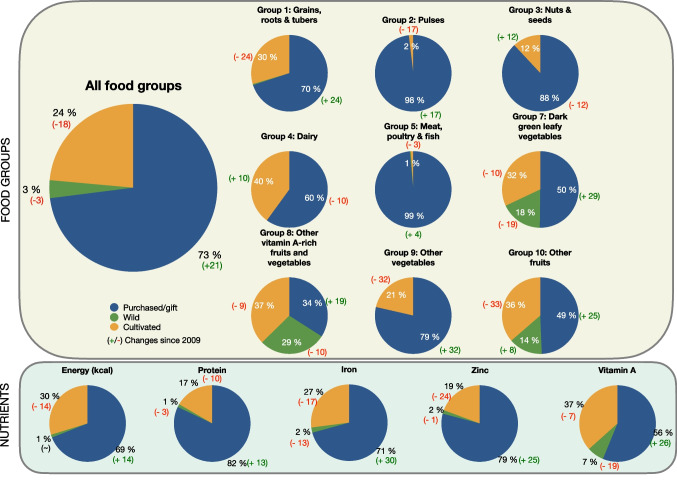
Sources of foods by food groups and nutrients. The figure shows the percentage of food items and nutrients sourced from either wild landscapes, cultivated land or purchased/obtained as gifts during wet season 2022 (N = 212 women of reproductive age). The ± shows the changes in percentage since 2009 (N = 184)

We used our 24 hours dietary recall data from 2009 and 2021/2022 to describe main changes in the general diet profile of respondents. That is, based on the recorded quantities of all foods consumed within the past 24 hours, we estimated the intake of calories and protein as well as micronutrients (vitamin A, iron, zinc). First, we converted all serving size measures (spoons, cups, plates, bowls, and photo aids) into grams. Then we used the Tanzanian Food Composition Table (Lukmanji & Hertzmark, 2008) to obtain the calorie, protein and micronutrient content in all foods consumed. In case a given food item was not included in the Tanzanian table, we used Food Composition Tables from Kenya, Malawi, Zambia and West Africa - in that order (FAO, 2022). If a food product was not found in any of the Food Composition Tables, we used proxies (for example cow milk as a proxy for goat milk). Because each respondent was interviewed on two non-consecutive days within one week in both the dry and wet season (four times in total), we calculated the *usual* intake for each season with a Multiple Source Method (MSM). The MSM is characterized by a two-part shrinkage technique applied to residuals of two regression models, one for the positive daily intake data and one for the event of consumption (Tooze, 2020). All food items were grouped according to the food groups included in FAO’s guidelines on *Minimum Dietary Diversity for Women* (FAO & FHI 360, 2016) and the relative nutrient contribution from each food group was then summed (Table 2). We note that we only compare dietary data from the wet seasons in 2009 and 2022, as we did not have access to quantitative intake data from the dry season 2009. Since we collected information on food sources (wild, cultivated, purchased/gift) for each food item, we were able to estimate the share of these sources for each of the food groups (Fig. 4). Moreover, we estimated the relative nutrient contribution from each of the three sources (Fig. 4).

**Table 2:** Percentage consuming the food group and mean relative contribution from each food group to overall diet in percentage of total amount of the 10 food groups in 2022. The (+/-) shows the difference from 2009 (N=184) to 2022 (N=212) (wet seasons only). Note that the 10 food groups do not cover total food intake as beverages, sodas, sweets, condiments and seasoning are not included

		Mean				
Food group	Percentage consuming the food group	Energy	Protein	Iron	Zinc	Vitamin A
1. Grains, white roots and tubers, and plantains	100 (~)	80.3 (-0.2)	48.3 (+5.3)	72.2 (+32.8)	61.1 (-0.4)	13.2 (-4.5)
2. Pulses (beans, peas and lentils)	24.1 (-3.6)	2.5 (-0.6)	4.2 (-1.8)	6.7 (+0.7)	5.2 (-1.9)	1.5 (+1.2)
3. Nuts and seeds	0.1 (-4.5)	0.0 (-0.5)	0.0 (-0.8)	0.0 (-0.5)	0.0 (-0.9)	0.0 (~)
4. Dairy	2.4 (-6.6)	0.1 (-0.6)	0.2 (-1)	0.0 (~)	0.1 (-1)	0.1 (-1.1)
5. Meat, poultry and fish	74.5 (-18.7)	10.8 (+2.9)	41.6 (+1.9)	10.2 (+1.2)	27.8 (+7.3)	26.5 (+17.9)
6. Eggs	0.0 (-0.2)	0.0 (~)	0.0 (~)	0.0 (~)	0.0 (~)	0.0 (~)
7. Dark green leafy vegetables	37.7 (-29.1)	2.2 (+0.6)	3.2 (-3)	7.7 (-33.9)	2.6 (-2.3)	42.5 (-17.1)
8. Other vitamin A-rich fruits and vegetables	34.4 (+18.8)	1.3 (+0.7)	0.7 (+0.3)	0.6 (-0.1)	0.9 (+0.3)	14.3 (+5.1)
9. Other vegetables	81.6 (+70.2)	0.1 (~)	0.1 (-0.2)	0.1 (-1.1)	0.1 (-0.4)	0.7 (-0.9)
10. Other fruits	50.0 (-27.7)	2.4 (-2.6)	1.3 (-1.4)	1.9 (0.4)	2.0 (-1.1)	1.1 (-0.6)

To compare changes in acquisition and consumption of wild foods over time, our dietary outcome variables of interest were: 1) Whether or not the woman consumed wild foods in the past seven days, and 2) the diversity of wild foods consumed and their sources (forest, farmland, purchased, other). These questions were asked separately from the 24 hours dietary recall. Our definition of wild foods, as ‘plants or animals that live and grow spontaneously, instinctively and *locally* in nature’, excluded fish caught in the ocean. If our respondents mentioned foods that were not typically considered to be wild (e.g., plantain), we would ask follow up questions to ensure that the food item matched our definition.

To estimate standards of living, we created a deprivation profile for each respondent based on the Global Multidimensional Poverty Index (MPI) using a gradient from 1 (deprived) to 0 (not deprived) (Alkire et al., 2021). We focused on the living standard dimension of the MPI (cooking fuel, sanitation, drinking water, electricity, housing, and assets). Aggregated MPI living standard values were also calculated for each village (Fig. 3).

We fitted a binomial general linear model (GLM) regression in R (version 4.3.2) to test whether consumption of wild foods (as well as consumption of purchased wild foods) in the past seven days (yes/no) was associated with village location, MPI living standards, age of the respondent, weekly food expenditures, number of trips to the forest in the past seven days, number of crops cultivated, and number of tree species on the farm. All correlation coefficients between the covariates were < 0.5 and the variance inflation factor (VIF) did not exceed a value of 5.

## Results

### Main changes in dietary profile

General food consumption was characterized by a dominance of staples, especially maize and cassava. Both maize and cassava is most often served as a stiff porridge (*ugali*)*,* with additional side dishes of dried sardines (*dagaa*) and cooked leafy vegetables (*mboga*). This very traditional style of serving is part of everyday diets in most households and has not changed considerably during the study period. For example, during wet season in 2022 all respondents consumed ‘grains, roots and tubers’ within the past 24 hours. In both wet seasons of 2009 and 2022, this food group was the main source of calories, protein and zinc, while the food group ‘dark green leafy vegetables’ was the main source of vitamin A.

Between 2009 and 2022, the percentage of people consuming meat, poultry and fish, and dark green leafy vegetables declined from 93.2% to 74.5% and from 66.8% to 37.7% respectively, while the percentage consuming other vegetables increased from 11.4% to 81.6%. In 2009, dark green leafy vegetables was the main source of iron with a relative contribution of 41.6% among the 10 food groups. In 2022, dark green leafy vegetables only contributed with 7.7% to the combined intake of iron from the 10 food groups (Table 2). Instead, the majority of iron came from grains, white roots and tubers, and plantains. Though dark green leafy vegetables were still the main source of vitamin A in 2022, its relative contribution had decreased from 59.6% to 42.5%. Another noticeable change is the increase in the share of zinc and vitamin A coming from meat, poultry, and fish (primarily sardines and other small, dried fish).

We observed a shift towards a greater reliance on purchased food items from the between wet season in 2009 to the wet season in 2022. In 2009, half (52%) of the food items (from the 10 food groups) were purchased. In 2022, this number had increased to 73% (Fig. 4). This increased reliance on market foods happened at the expense of cultivated and wild foods, which decreased with 18% and 3%, respectively. Especially dark green leafy vegetables and other vitamin A-rich fruits and vegetables from the wild decreased, with 19% and 10%, respectively. The largest degree of marketization occurred in food groups covering fruits, vegetables, pulses, grains, roots and tubers.

Despite these changes, a similar pattern of food sourcing across food groups was observed across study rounds. In both 2009 and 2022, pulses as well as meat, poultry and fish were predominantly purchased, while dark green leafy vegetables, other vitamin-A rich fruits and vegetables, and other fruits came from multiple sources.

We found an increase in the relative contribution of purchased products to the intake of calories, protein, vitamin A, zinc, and iron (Fig. 4). This came at the expense of wild foods and cultivated foods. That is, in 2022 wild food sourcing only contributed with 2% of total iron intake as compared to 15% in 2009 and 7% of total vitamin A intake as compared to 26% in 2009. In 2022, purchased foods contributed with 69% of total calorie intake as compared to 55% in 2009 and 82% of total protein intake as compared to 69% in 2009.

### Changes in consumption, diversity, and sources of wild foods

**Fig. 5:**
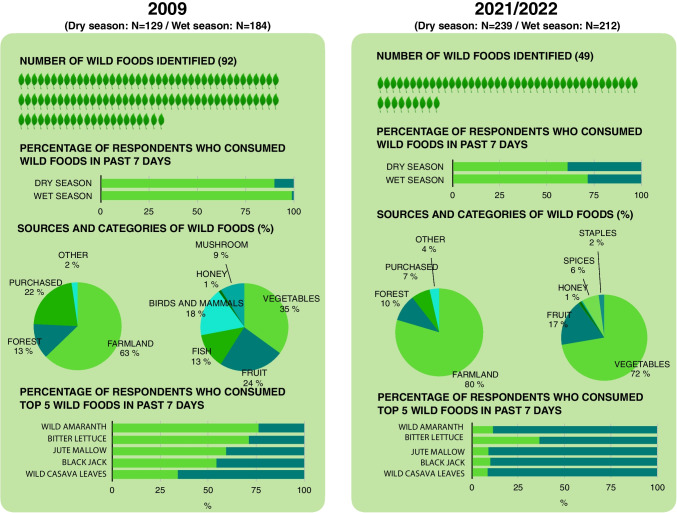
Changes in wild food consumption from dry and wet seasons in 2009 to 2021/2022. The figure shows how the frequency and diversity of wild food consumption has declined over the period, and how wilds foods are increasingly sourced from farmlands. Note that the food sources in this figure differ from Fig. 4 (cultivated, purchased/gifts and wild) since this figure shows where only *wild* foods are sourced. Farmland is thus not to be compared directly to cultivated as wild foods can be sourced from farmland although they are not cultivated. The figure shows mean values across dry and wet seasons (except for the top bar chart on ‘percentage of respondents who consumed wild foods in past 7 days’)

Our results show a substantial decline in wild food consumption from 2009 to 2021/2022 across both dry and wet season. That is, 98% of the respondents reported to have consumed wild foods within the last seven days in the wet season of 2009 as compared to only 72% in 2022. The decline was even larger when looking at the dry season (93% in 2009 as compared to 61% in 2021) (Fig. 5).

Our results also show a decline in the diversity of wild foods consumed from 2009 to 2021/2022: From 92 to 49 different types of wild foods. Examples of wild foods that became less frequently consumed are wild amaranth, jute mallow, black jack, wild cassava leaves, crab fish and cane rats. The mean number of species consumed per woman within the past seven days declined from four in 2009 to one in 2021/2022. Fruits, vegetables, and spices were the main wild foods consumed in 2021/2022, whereas people consumed additional wild food groups, such as wild fish, wild birds and mammals and wild mushrooms, in 2009. The majority of wild foods were collected and gathered in farmlands (63% in 2009, 80% in 2021/2022), but people also purchased wild foods (22% in 2009, 7% in 2021/2022) and collected them in the forest (13% in 2009, 10% in 2021/2022). The five wild foods consumed most frequently by respondents across dry and wet season in both 2009 and 2021/2022 were wild amaranth, bitter lettuce, jute mallow, black jack and wild cassava leaves – all being different types of dark green leafy vegetables. These species either thrive as weeds in farmland and fallows in the wet season or can be found in the forest in the dry season. The consumption of these species has declined substantially. For example, wild amaranth was consumed by 76% of the respondents in 2009 but only 11% in 2022 (Fig. 5). Analogously, the consumption of less common species, such as vine spinach decreased from being consumed by 26% of the respondents in 2009 to 6% in 2021/2022, which translates into a decline in the total number of wild foods reported. When using binomial GLM regression to test if village location, MPI level, age of respondent, food expenditures, number of trips to the forest, number of crops cultivated, and number of tree species on the farm was associated with whether or not the respondent had consumed wild foods in the past seven days, no significant associations were observed. The focus groups, described below, did not reveal noticeable differences between villages either, except for restricted forest access being a more pronounced issue in Shambangeda — the study village most adjacent to the forest reserve owned by the central government.

### Local perceptions of drivers of wild food consumption

The participants described five main tendencies in the changed wild food consumption from 2009 to 2021/2022: 1) Declining availability of wild foods, 2) increased wild food sourcing from farmlands rather than forests, 3) less access to wild foods (both de facto and de jure), 4) increased commercialization of wild foods, and 5) new food preferences towards processed and market-based foods.

#### Declining availability: There are no fish in the river anymore

Even in the 2009 study period, focus group participants were reporting a decline in wild food availability and very limited collection of foods from the forest (e.g., noting “there are no fish in the river any more” and that it had been a long time since there was enough wild pigs to make it worthwhile to pursue hunting). Many people reported that deforestation and population growth had already reduced the availability of wild foods. Also, in 2021/2022 participants mentioned that it was very difficult to find wild vegetables and that the ones collected were mostly growing around the farms*.* Participants reported a decrease in the availability of all wild animals as well as specific wild fruits and vegetables, with the main reasons being lack of rainfall, warmer climate, increased deforestation, and loss of wild habitats.

#### From forest to farmlands

In the 2009 study period even people in communities with a high degree of forest cover talked about the forest as being far away and hard to get to. Participants explained that if the forest was closer, people would go there to get food. Participants reported time constraints as a key factor for not going to the forest to collect wild foods, except in Shambangeda (the community adjacent to the forest reserve owned by the government):

*“[The forest] is not very important to us, it doesn’t benefit us much… we only consider it important for firewood.*”

Focus group participant in 2009, Shambangeda

During the focus groups in 2021/2022, participants explained the diminishing role of the forest as a source of wild foods. The participants described walking to the forest as a long and steep trek (mean walking time to the natural forests was 118 min but ranging from 20–300 min), and they portrayed the forest as dense and impenetrable when they finally got there. Most people only went if no other alternatives were available. Instead, several participants transplanted wild vegetables from forests to farmlands to improve convenience and save time. As one participant in Shambangeda explained:

*“Some wild foods are more accessible today as compared to 10 years ago because today they grow around our farms and houses. Before we had to go to the forest to find them.”*

Focus group participant in 2022, Shambangeda

The shift from forest to farmland as a source of wild food was also reflected in everyday cooking, where vegetables such as bitter lettuce, black jack and nightshade from farmlands and homegardens were commonly used in relishes.

#### Less access to wild foods: Life was easy when the forest was open

A key theme in 2009 was a lack of access to wild foods because of de facto and de jure barriers. Collection and acquisition of wild foods in Tanzania is regulated and managed on different levels. The Ministry of Natural Resources and Tourism sets legislations through its National Forest Policy (United Republic of Tanzania, 1998), National Forest Act (United Republic of Tanzania, 2002), and National Forest Regulations (United Republic of Tanzania, 2008). The Ministry of Natural Resources also sets the national regulatory frame on hunting of wild animals through the Wild Life Conservation Act, the National Wild Life Policy, and the Wild Life Conservation Regulations (United Republic of Tanzania, 2022). The latter has imposed strict decrees on any type of wildlife hunting in forest reserves, and might explain the observed decline in consumption of wild animal foods in our study sites. Also, wild animals are not allowed to be hunted in private farms without a permit. However, there is lenience in implementation of the regulation when it comes to rodents (e.g., can rats). In 2009 participants reported *“life was easy when the forest was open”* (i.e., before the implementation and enforcement of forest protection). In addition to these de jure barriers to access, participants also described de facto barriers including: A perceived threat of harassment from forest guards, fear of running into people engaged in illegal activities in the forest, and fear of violence or sexual assault if people were in the forest alone. This theme of legal access was ranked as very important in focus group ranking exercises. Already in 2009 focus group participants reported major declines in wild meat availability, confirming survey results where very little wild meat consumption was reported. However, we note that bush meat use may be underreported since it is a highly sensitive topic associated with illegal poaching activities (van Vliet et al., 2022).

Despite that lack of access was already mentioned in 2009 as a recurring issue, focus group participants in 2021/2022 noted that access to forests for the collection of wild foods had decreased even further due to imposed restrictions. A recent amendment to the National Forest Regulations (United Republic of Tanzania 2017) introduced forest entrance fees and fees for collection of non-timber forest products in all government owned and managed forests. The four villages in this study represent four different forest management systems; 1) government managed monoculture forest plantations (Bombani), 2) government managed natural forests (Shambangeda), 3) community managed natural forests (Kiwanda), and 4) individual managed woodlots (Tongwe). As such, changes in forest regulations differed from village to village. Despite the different forest management systems, we found no significant differences in the number of respondents reporting wild food consumption within the past seven days across the four villages. Across all four villages, the respondents and the focus group participants mentioned the enforcement of wildlife regulation as a reason for the decline in consumption of wild animals. As one of the respondents explained:

“*We ate meat more often when we could catch wild animals. There are still some people who go to hunt in the forest but it is not legal and they will be punished if they are caught.”*

Focus group participant in 2021, Kiwanda

#### Increased commercialization: If you want wild foods you need to buy them

Focus group participants in all four villages described how they had become more reliant on market-based foods, especially maize flour and rice, and less dependent on traditional foods, such as wild leafy vegetables. This was confirmed by our survey data, showing how the share of purchased food items increased from 52 to 73% between 2009 and 2021/2022 (Fig. 4). They described this transition as driven by a) better market access due to more motorbikes in the area, and b) higher incomes due to increased cash crop production (e.g., black pepper, cardamom, cloves and teak). None of the focus group participants mentioned changes in food prices as causing a decline in wild food consumption. On the contrary, in two of the four focus groups, some participants stated that consumption of specific wild foods had recently increased because people were searching for cheaper alternatives to market foods. The participants explained how their income opportunities had decreased due to harvest failure of cash crops and how that had led them to collect wild foods. As one respondent said:

*“Now, as income is low, we go back to eating the traditional foods that we used to eat before. This means that we eat more wild foods these days because we do not need to pay for them.”*

Focus group participant in 2021, Bombani

Participants reported an increased commodification of wild foods, with wild foods increasingly purchased from vendors and at market. With declining wild food availability in the forest, participants reported that some wild foods (e.g., jute mallow) were now purchased or people switched to non-wild vegetable species, such as cassava leaves. Half of all dark green leafy vegetables were purchased in 2021/2022 as compared to only 21% in 2009 (Fig. 4). In Shambangeda, one woman said:

*“If you want to have wild food, you need to buy it at the market where people from other villages come and sell it.”*

Focus group participant in 2021, Shambangeda

With the increased commodification, one might expect that wild food purchases would be most prevalent among people with higher purchasing power. Yet, we found that households’ living standard was not associated with the consumption of wild food (nor the consumption of purchased wild food) (based on our binomial GLM models using the consumption of wild foods as well as consumption of purchased wild food as outcome variables). This indicates that wild food consumption is not restricted to those households that are better off.

#### New food preferences: The world has become a village

When asked about food preferences, some participants expressed how they preferred to eat processed foods from the market rather than wild ‘free’ foods from forests or surrounding landscapes. Our focus groups revealed an intergenerational difference in taste preferences and attitudes towards wild foods. In general, the elders were able to name and describe many wild foods that the younger generation was not familiar with and in some cases had not even tasted (especially wild animals). Also, wild dark green leafy vegetables in Tanzania are often characterized by a bitter taste that the younger generations did not appreciate. Instead, the younger generations would express higher preferences towards processed market-based foods. Even in 2009, the more elderly participants suggested that young people did not like to eat wild foods and traditional vegetables; some of the elders also indicated that many young people were lazy or lacked motivation to go to the forest to look for wild foods. In 2022, a young woman stated:

*“The world has become a village. We can see how other people eat in the cities and we want the same kind of foods. We prefer the processed and refined flour instead of mayugwa (local tuber) and we want apples instead of jack fruits. That is why many young people are not eating traditional wild foods any longer”*

Focus group participant in 2022, Kiwanda

In this specific focus group, an elderly woman responded to the young woman:

*“But when you go to the hospital, the doctor will tell you to stop eating processed food and eat the wild vegetables instead.”*

Focus group participant in 2022, Kiwanda

For the people who did consume wild foods, specific wild foods were preferred over others due to their taste.

## Discussion and conclusions

### Food environment changes affect wild food consumption

Below we fit the information from the narratives above into the different dimensions of the food environment framework. We note that it is challenging to fit complex real-life scenarios into the highly compartmentalized dimensions of the food environment frameworks. Also, it is difficult to quantitatively assess the relative importance of the different food environment dimensions in terms of their influence on food choice. Finally, we suggest a new dimension to better capture wild foods in a food environment framework approach.

#### Availability

Although the availability dimension is given little attention in food environment studies focused on urban and higher income areas, we found reduced wild food availability to be one of the key factors listed by communities as driving consumption choices.

The main reasons for the reduced availability were, according to the participants, climatic changes, deforestation, and loss of biodiversity caused by farmland expansion. This is in line with other studies: A recent assessment of 1039 wild plant and mushroom food species in the IUCN Redlist found that 63% were decreasing in abundance (Pilling & Bélanger, 2019). And a recent review of 78 studies on wild plants found that most communities experienced a decline in the availability of wild plants and mushrooms - and that communities perceived land use change and over-exploitation to be the main drivers of the decline (affecting 38 and 31% of taxa respectively) (Schunko et al., 2022). Moreover, a recent case study from Laos found that declining consumption of forest foods was perceived to be caused by deforestation and forest degradation (Jendrensen & Rasmussen, 2022).

As such, the availability dimension is likely more relevant in places with limited market access or poorly functioning markets. We also observed that the boundary between “availability” and “accessibility” is blurry as it remains unclear how far away a given wild food can be in order to still be “available” to a community?

#### Access and convenience

Differentiating access from convenience is likewise challenging. Both dimensions are largely measured in terms of time (to collect/harvest or prepare wild foods). In this study, few participants noted cooking or processing time as shaping their food choice, but they did note the “convenience” of having wild vegetables close to the home (in the fields and homegardens). The scarcity of certain species has led to reduced access and convenience, causing communities to a) shift from forests to farmlands when collecting these foods, b) purchase wild foods in the market, and c) shift to non-wild alternatives. These changes suggest a shift from the natural food environments to informal built food environments. Also, they reflect the complexity of dietary choice and the multiple trajectories of dietary transitions when wild foods become less available/accessible.

#### Price and affordability

Participants reported price and affordability as important drivers of their general food choices and whether they pursued wild food consumption. In 2009, people reported that as soon as a given household had some income, the household would purchase more food. In 2021/2022, people reported that lower consumption of wild foods was in part due to improved incomes, better market access, and with market foods being more affordable. The fact that people continued to purchase wild foods despite rising prices suggests that wild foods were still preferred (primarily by the elderly) - and that access to wild foods is a pronounced barrier for continued consumption.

#### Food properties, desirability, and food preferences

Among our participants, food properties in terms of quality, safety or contamination did not appear to be a driver of food choices. Yet, cultural preferences appeared to fuel continued wild food consumption, especially among the elder participants. The focus groups revealed intra-generational differences in food preferences, with younger participants expressing aspirations for more ‘modern’ lifestyles, where foods such as fried chicken, frites and soda water were valued higher than traditional vegetables. This is in line with other recent studies: A study from Uganda found that declining wild food use was associated with intra-generational erosion of local knowledge on foraging, increased cultivation and loss of wild habitats (Ekesa et al., 2022). And in Indonesia, declining wild food consumption was linked to low availability, time constraints, limited knowledge of the nutritional value and a “change of taste” among younger generations (Pawera et al., 2020).

 The role of food as a marker of identity is well-explored in the social sciences (Fischler, 1988), if not well developed in the food environment. The role of wild foods in marking cultural belonging which in turn can drive food choices has previously been established in Tanzania (Powell et al., 2017).

#### Marketing and regulation

Participants did not mention exposure to marketing or regulations (of market foods) as a driver of food choices. However, even in rural communities, people are exposed to marketing—from ubiquitous Coca-Cola signs to radio and TV adds. Thus, it seems unlikely that the marketing, predominantly of highly processed foods (e.g., sweets, snacks, seasonings, instant noodles) is not affecting dietary choice in a more subconscious manner (Marteau et al., 2012).

### Legal access to wild resources as a new dimension

Based on our findings, we suggest a new dimension to the food environment frameworks: *Legal access to wild resources*. We do so because legal access to wild resources was mentioned as a key driver behind food choices in focus groups in both 2009 and 2021/2022. While the personal ‘access’ dimension captures whether people have the time and energy required to collect or hunt wild foods, there is currently no dimension in the food environment frameworks capturing whether restricted legal access to wild resources might act as a barrier for certain food choices (e.g., wild food consumption). Issues related to legal access often play out at the external and structural level (e.g., forest and wildlife regulation, privatization, land reforms, conservation initiatives), but, as became evident in the focus group discussions, these issues can also play out at the local level, for example in the form of threats to access such as harassment from forest guards.

Even though restricted forest access was mentioned in all focus groups as a reason behind decreased wild food consumption – especially in Shambangeda, where people live in near proximity to the forest reserve, we did not find any significant differences in wild consumption across the four study sites (representing different forest management systems). One possible explanation is the increased consumption of wild foods sourced around farmland which potentially makes up for the decreased access to wild forest foods (Fig. 5). Yet, we note that disentangling the push (less access to wild foods) vs the pull effect (more access to alternatives) has been one of the more challenging aspects of this study, as it appears that people are better able to articulate the push compared to the pull.

**Fig. 6:**
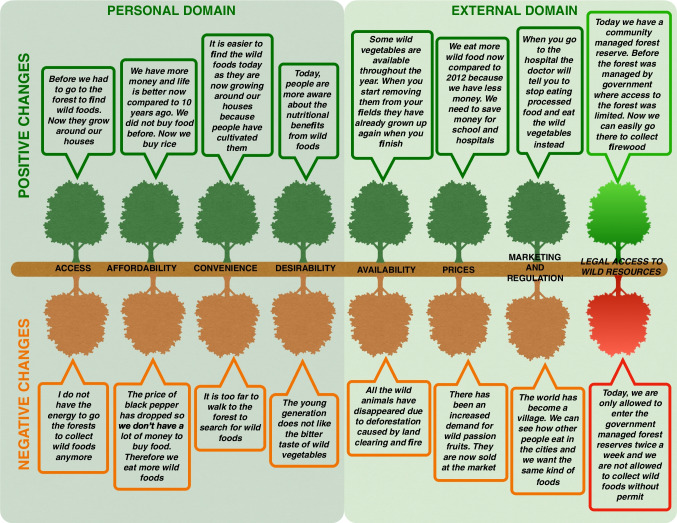
Using the food environment framework to examine changes in wild food consumption 2009–2022. The figure shows quotes from focus group participants on how each of the external and personal factors were perceived to have affected local wild food consumption in both negative and positive directions from 2009–2021/2022. The figure does not include ‘Vendor and Product Properties’ since this dimension was not mentioned by the respondents. The bright green and red boxes on the far right depict a new dimension that we suggest: ‘Legal access to wild resources’

 In summary, by including a new dimension of “legal access to wild resources”, we can capture access to wild foods (and other natural resources such as fuelwood). This new dimension would be well placed in the “external domain” according to Turner et al.'s (2017 p. 4) definition: *“A domain that relates to the world of opportunities and constraints that are ‘out there’ within a given context”* (Fig. 6).

### Conceptual advancements of the food environment framework

Our findings have three conceptual implications for the food environment framework.

First, the framework proved helpful in its ability to provide an overview of possible food sources for individuals at a point-in time. However, the framework does not provide efficient analytical tools to examine seasonal variation nor changes over longer time spans as the factors that drive dietary change remain vaguely articulated by participants. The analytical containers of the framework (domains and dimensions) invite for static categorizations rather than analysis of elasticity and transformations over time. The interrelationships between some domains and dimensions (e.g., access and availability) can at times be blurred, which also makes it challenging to identify shifts between domains and dimensions over time. We suggest more emphasis is placed on longitudinal changes to assess how changes translate into shifting food environments. The food environment transition model (Downs et al., 2020) provides an analytical tool for cross-sectional studies and long-term changes (transition from agrarian to urban stages of societies). We have found the approach used by Downs et al. (2020) to be helpful in our case study, but more work is needed to develop food environment frameworks that can examine season variations and longitudinal changes occurring within the same transition stage.

Second, the Downs et al. (2020) framework clearly distinguishes between wild versus cultivated food sources and between natural versus built food environments. Our results suggest that these demarcations are not easily defined in real life settings. Some foods (e.g., wild amaranth) are found across a range of systems, including forests, semi-wild/managed environments, homegardens, and farmlands (see also Bharucha & Pretty, 2010). Moreover, wild amaranth from any of these sources can be found in informal markets. Our case study suggests that rather than relying on separate “food environments”, people are procuring foods from multiple sources within one mixed food environment. Dietary transitions, for example from wild leafy vegetables to purchased (cultivated) leafy vegetables, are driven by both push factors (wild food becoming less available and accessible or restricted due to changes in legal access) and pull factors (improved affordability of purchased food). We find it more conducive to think about these factors as part of a single food system as it undergoes transition. The food environment framework was also not fully able to capture the complex dynamics and trade-offs that shape people’s consumption of wild, cultivated and market-based foods. For example, it remains difficult to determine if reduced access and low convenience act to “push” dietary choices away from traditional wild foods, or if higher convenience and desirability of purchased options “pull” people towards purchased and cultivated foods. Disentangling the role of the food environment in transitions away from wild foods often suffers from the blurry distinction between cultivated and wild foods – something that is further complicated when the distinction changes over time.

Third, we have proposed an additional dimension in the food environment frameworks: “Legal access to wild resources” covering access to wild foods (and other natural resources such as fuel wood).

In summary, food environment research could be strengthened by focusing on temporal transitions between wild, cultivated and formal- and informal market-based food sources. Also, it would be useful to further explore how these sources provide different foods and nutrients, and how their relative importance changes over time. Because rural food environments in LMICs are highly rooted and shaped by surrounding landscapes, future research needs to link landscape dynamics with food environment domains - and attend to how these linkages in turn impact nutrient intake locally.

## Data Availability

Data are available upon request. Please contact the lead author (rso@ign.ku.dk).

## References

[CR1] Akombi, B. J., Agho, K. E., Merom, D., Renzaho, A. M., & Hall, J. J. (2017). Child malnutrition in sub-Saharan Africa: A meta-analysis of demographic and health surveys (2006–2016). *PLoS ONE,**12*(5), e0177338. 10.1371/journal.pone.0177338 28494007 10.1371/journal.pone.0177338 PMC5426674

[CR2] Alkire, S., Kanagaratnam, U., & Suppa, N. (2021). The global multidimensional poverty index (MPI) 2021. *OPHI MPI Methodological Note 51. *Oxford Poverty and Human Development Initiative, University of Oxford.

[CR3] Asprilla-Perea, J., & Diaz-Puente, J. M. (2019). Importance of wild foods to household food security in tropical forest areas. *Food Security,**11*(1), 15–22. 10.1007/s12571-018-0846-8

[CR4] Bailey, R. L., West, K. P., Jr., & Black, R. E. (2015). The epidemiology of global micronutrient deficiencies. *Annals of Nutrition and Metabolism,**66*(Suppl. 2), 22–33. 26045325 10.1159/000371618

[CR5] Bezner Kerr, R., Hasegawa, T., Lasco, R., Bhatt, I., Deryng, D., Farrell, A., Gurney-Smith, H., Ju, H., Lluch-Cota, S., Meza, F., Nelson, G., Neufeldt, H., & Thornton, P. (2022). Food, fibre, and other ecosystem products. *Climate Change 2022: Impacts, Adaptation and Vulnerability. Contribution of Working Group II to the Sixth Assessment Report of the Intergovernmental Panel on Climate Change* (pp. 713–906). 10.1017/9781009325844.007

[CR6] Bharucha, Z., & Pretty, J. (2010). The roles and values of wild foods in agricultural systems. *Special Issue: Food Security: Feeding the World in 2050,**365*(1554), 2913–2926. 10.1098/rstb.2010.0123 10.1098/rstb.2010.0123 PMC2935111 20713393

[CR7] Bhushi, K. (2021). Hunger and pandemic: Wild edibles as future of food. *Society and Culture in South Asia,**7*(1), 163–168. 10.1177/2393861720977404

[CR8] Blaney, S., Beaudry, M., & Latham, M. (2009). Contribution of natural resources to nutritional status in a protected area of Gabon. *Food and Nutrition Bulletin,**30*(1), 49–62. 10.1177/156482650903000105 19445259 10.1177/156482650903000105

[CR9] Blundo-Canto, G., Cruz-Garcia, G. S., Talsma, E. F., Francesconi, W., Labarta, R., Sanchez-Choy, J., et al. (2020). Changes in food access by mestizo communities associated with deforestation and agrobiodiversity loss in Ucayali, Peruvian Amazon. *Food Security,**12*(3), 637–658. 10.1007/s12571-020-01022-1

[CR10] Borelli, T., Hunter, D., Powell, B., Ulian, T., Mattana, E., Termote, C., et al. (2020). Born to eat wild: An integrated conservation approach to secure wild food plants for food security and nutrition. *Plants,**9*(10), 1–37. 10.3390/plants9101299 10.3390/plants9101299 PMC7601573 33019632

[CR11] Burgess, N., Doggart, N., & Lovett, J. C. (2002). The Uluguru Mountains of eastern Tanzania: The effect of forest loss on biodiversity. *Oryx,**36*(2), 140–152. 10.1017/S0030605302000212

[CR12] Campbell, D., Moulton, A. A., Barker, D., Malcolm, T., Scott, L., Spence, A., et al. (2021). Wild food harvest, food security, and biodiversity conservation in Jamaica: A case study of the Millbank Farming Region. *Frontiers in Sustainable Food Systems*. 10.3389/fsufs.2021.663863

[CR13] Capitani, C., van Soesbergen, A., Mukama, K., Malugu, I., Mbilinyi, B., Chamuya, N., et al. (2019). Scenarios of land use and land cover change and their multiple impacts on natural capital in Tanzania. *Environmental Conservation,**46*(1), 17–24. 10.1017/S0376892918000255

[CR14] Carignano Torres, P., Morsello, C., Orellana, J. D. Y., Almeida, O., de Moraes, A., Chacón-Montalván, E. A., et al. (2022). Wildmeat consumption and child health in Amazonia. *Scientific Reports,**12*(1), 5213. 10.1038/s41598-022-09260-3 35388037 10.1038/s41598-022-09260-3 PMC8986765

[CR15] Carletto, C., Corral, P., & Guelfi, A. (2017). Agricultural commercialization and nutrition revisited: Empirical evidence from three African countries. *Food Policy,**67*, 106–118. 10.1016/j.foodpol.2016.09.020 28413250 10.1016/j.foodpol.2016.09.020 PMC5384450

[CR16] Ceppi, S. L., & Nielsen, M. R. (2014). A Comparative Study on Bushmeat Consumption Patterns in ten Tribes in Tanzania. *Tropical Conservation Science,**7*(2), 272–287. 10.1177/194008291400700208

[CR17] Chegere, M. J., & Stage, J. (2020). Agricultural production diversity, dietary diversity and nutritional status: Panel data evidence from Tanzania. *World Development,**129*, 104856. 10.1016/j.worlddev.2019.104856

[CR18] Constantinides, S. V. (2021). Using a global food environment framework to understand relationships with food choice in diverse low- and middle-income countries. *Global Food Security, 16, *100511.

[CR19] Cooper, M., Zvoleff, A., Gonzalez-Roglich, M., Tusiime, F., Musumba, M., Noon, M., et al. (2018). Geographic factors predict wild food and nonfood NTFP collection by households across four African countries. *Forest Policy and Economics,**96*, 38–53. 10.1016/j.forpol.2018.08.002 30393458 10.1016/j.forpol.2018.08.002 PMC6191931

[CR20] Dewey, K. G. (1989). Nutrition and the commoditation of food systems in Latin America and the Caribbean. *Social Science & Medicine,**28*(5), 415–424. 10.1016/0277-9536(89)90097-X 2648596 10.1016/0277-9536(89)90097-x

[CR21] Downs, S. M., Ahmed, S., Fanzo, J., & Herforth, A. (2020). Food environment typology: Advancing an expanded definition, framework, and methodological approach for improved characterization of wild, cultivated, and built food environments toward sustainable diets. *Foods,**9*(532), 32. 10.3390/foods9040532 PMC7230632 32331424

[CR22] Ekesa, B., Fongar, A., & Nasser, M. (2022). Access to and Utilization of Wild Species for Food and Nutrition Security in Teso and Acholi Sub-regions of Uganda. *Frontiers in Sustainable Food Systems,**6*, 836212. 10.3389/fsufs.2022.836212

[CR23] Fa, J. E., Olivero, J., Real, R., Farfán, M. A., Márquez, A. L., Vargas, J. M., et al. (2015). Disentangling the relative effects of bushmeat availability on human nutrition in central Africa. *Scientific Reports*, *5*(1), 8168. 10.1038/srep08168 10.1038/srep08168 PMC4313087 25639588

[CR24] FAO. (2016). *Influencing food environments for healthy diets* (p. 154). Rome: Food and Agriculture Organization of the United Nations.

[CR25] FAO. (2021). *World Food and Agriculture – Statistical Yearbook 2021*. FAO. 10.4060/cb4477en

[CR26] FAO. (2022). *International Network of Food Data Systems (INFOODS)* (Food Composition Tables). https://www.fao.org/infoods/infoods/tables-and-databases/africa/en/. Accessed 10 October 2022.

[CR27] FAO, & FHI 360. (2016). *Minimum Dietary Diversity for Women- A Guide to Measurement*. Rome: FAO.

[CR28] FAO, IFAD, UNICEF, WFP, & WHO. (2022). The State of Food Security and Nutrition in the World 2022. Repurposing food and agricultural policies to make healthy diets more affordable. Rome: FAO. 10.4060/cc0639en

[CR29] Fischler, C. (1988). Food, self and identity. *Social Science Information,**27*(2), 275–292. 10.1177/053901888027002005

[CR30] Fleuret, A. (1979). The role of wild foliage plants in the diet. *Ecology of Food and Nutrition,**8*(2), 87–93. 10.1080/03670244.1979.9990549

[CR31] Fungo, R., Muyonga, J., Kabahenda, M., Kaaya, A., Okia, C. A., Donn, P., et al. (2016). Contribution of forest foods to dietary intake and their association with household food insecurity: A cross-sectional study in women from rural Cameroon. *Public Health Nutrition,**19*(17), 3185–3196. 10.1017/S1368980016001324 27265306 10.1017/S1368980016001324 PMC10270934

[CR32] Golden, C. D., Fernald, L. C. H., Brashares, J. S., Rasolofoniaina, B. J. R., & Kremen, C. (2011). Benefits of wildlife consumption to child nutrition in a biodiversity hotspot. *Proceedings of the National Academy of Sciences,**108*(49), 19653–19656. 10.1073/pnas.1112586108 10.1073/pnas.1112586108 PMC3241784 22106297

[CR33] Guyu, D. F., & Muluneh, W.-T. (2015). Wild foods (plants and animals) in the green famine belt of Ethiopia: Do they contribute to household resilience to seasonal food insecurity? *Forest Ecosystems,**2*(1), 34. 10.1186/s40663-015-0058-z

[CR34] Hall, C. M., Rasmussen, L. V., Powell, B., Dyngeland, C., Jung, S., & Olesen, R. S. (2022). Deforestation reduces fruit and vegetable consumption in rural Tanzania. *Proceedings of the National Academy of Sciences,**119*(10), e2112063119. 10.1073/pnas.2112063119 10.1073/pnas.2112063119 PMC8915834 35238660

[CR35] Hall, J. C. (2021). Food security in the Era of COVID-19: Wild food provisioning as resilience during a global pandemic. *Culture, Agriculture, Food and Environment*. 10.1111/cuag.12275

[CR36] Herforth, A., & Ahmed, S. (2015). The food environment, its effects on dietary consumption, and potential for measurement within agriculture-nutrition interventions. *Food Security,**7*(3), 505–520. 10.1007/s12571-015-0455-8

[CR37] Hervas, A. (2020). Cultivating vulnerability: Oil palm expansion and the socio-ecological food system in the Lachuá Ecoregion, Guatemala. *Regional Environmental Change,**20*(2), 45. 10.1007/s10113-020-01630-9

[CR38] Hervas, A., & Isakson, S. R. (2020). Commercial agriculture for food security? The case of oil palm development in northern Guatemala. *Food Security,**12*(3), 517–535. 10.1007/s12571-020-01026-x

[CR39] Hickey, G. M., Pouliot, M., Smith-Hall, C., Wunder, S., & Nielsen, M. R. (2016). Quantifying the economic contribution of wild food harvests to rural livelihoods: A global-comparative analysis. *Food Policy,**62*, 122–132. 10.1016/j.foodpol.2016.06.001

[CR40] HLPE. (2020). Food security and nutrition: building a global narrative towards 2030. A report by the high level panel of experts on food security and nutrition of the committee on world food security. *Rome*, 112.

[CR41] Hua, J., Seto, E., Li, Y., & Wang, M. C. (2014). Development and evaluation of a food environment survey in three urban environments of Kunming, China. *BMC Public Health,**14*(1), 235. 10.1186/1471-2458-14-235 24602326 10.1186/1471-2458-14-235 PMC4016521

[CR42] Ickowitz, A., Powell, B., Rowland, D., Jones, A., & Sunderland, T. (2019). Agricultural intensification, dietary diversity, and markets in the global food security narrative. *Global Food Security,**20*, 9–16. 10.1016/j.gfs.2018.11.002

[CR43] Ickowitz, A., Rowland, D., Powell, B., Salim, M. A., & Sunderland, T. (2016). Forests, trees, and micronutrient-rich food consumption in Indonesia. *PLoS ONE,**11*(5), e0154139. 10.1371/journal.pone.0154139 27186884 10.1371/journal.pone.0154139 PMC4871346

[CR44] Jackson, K., & Bazeley, P. (2019). *Qualitative data analysis with NVivo*. Sage.

[CR45] Jendresen, M. N., & Rasmussen, L. V. (2022). The importance of forest foods for diet quality: A case study from Sangthong District, Laos. Trees, Forests and People, 7. https://doi.org/10.1016/j.tfp.2021.100166

[CR46] Jones, A. D. (2017). Critical review of the emerging research evidence on agricultural biodiversity, diet diversity, and nutritional status in low- and middle-income countries. *Nutrition Reviews,**75*(10), 769–782. 10.1093/nutrit/nux040 29028270 10.1093/nutrit/nux040 PMC5914317

[CR47] Kaya, H. O., & Lyana, A. (2014). Knowledge and perceptions of rural communities on wild food resources consumption in Tanzania. *Journal of Human Ecology,**48*(1), 53–60. 10.1080/09709274.2014.11906774

[CR48] Kc, K. B., Dias, G. M., Veeramani, A., Swanton, C. J., Fraser, D., Steinke, D., et al. (2018). When too much isn’t enough: Does current food production meet global nutritional needs? *PLoS ONE,**13*(10), e0205683. 10.1371/journal.pone.0205683 30352069 10.1371/journal.pone.0205683 PMC6198966

[CR49] Keding, G., Weinberger, K., Swai, I., & Mndiga, H. (2007). *Diversity, traits and use of traditional vegetables in Tanzania*. AVRDC-WorldVegetableCenter.

[CR50] Kennedy, G., Nantel, G., & Shetty, P. (2003). The scourge of" hidden hunger": global dimensions of micronutrient deficiencies. *Food Nutrition and Agriculture,* (32), 8–16.

[CR51] Khoury, C. K., Bjorkman, A. D., Dempewolf, H., Ramirez-Villegas, J., Guarino, L., Jarvis, A., et al. (2014). Increasing homogeneity in global food supplies and the implications for food security. *Proceedings of the National Academy of Sciences,**111*(11), 4001–4006. 10.1073/pnas.1313490111 10.1073/pnas.1313490111 PMC3964121 24591623

[CR52] Lukmanji, Z., & Hertzmark, E. (2008). *Tanzania food composition tables*. Dar es Salaam, Tanzania: MUHAS, TFNC and HSPH.

[CR53] Marteau, T. M., Hollands, G. J., & Fletcher, P. C. (2012). Changing human behavior to prevent disease: The importance of targeting automatic processes. *Science,**337*(6101), 1492–1495. 10.1126/science.1226918 22997327 10.1126/science.1226918

[CR54] Meemken, E.-M., & Bellemare, M. F. (2020). Smallholder farmers and contract farming in developing countries. *Proceedings of the National Academy of Sciences,**117*(1), 259–264. 10.1073/pnas.1909501116 10.1073/pnas.1909501116 PMC6955373 31836695

[CR55] Ministry of Health, Community Development, Gender, Elderly and Children (MoHCDGEC) [Tanzania Mainland], Ministry of Health (MoH) [Zanzibar], Tanzania Food and Nutrition Centre (TFNC), National Bu reau of Statistics (NBS), Office of the Chief Government Statistician (OCGS) [Zanzibar] and UNICEF, Tanzania Food and Nutrition Centre (TFNC, National Bureau of Statistics (NBS), Office of the Chief Government Statistician (OCGS), UNICEF. (2018). *Tanzania National Nutrition Survey using SMART Methodology (TNNS) 2018*. Dar es Salaam, Tanzania: MoHCDGEC, MoH, TFNC, NBS, OCGS, and UNICEF.

[CR56] Murray, S. S., Schoeninger, M. J., Bunn, H. T., Pickering, T. R., & Marlett, J. A. (2001). Nutritional composition of some wild plant foods and honey used by Hadza foragers of Tanzania. *Journal of Food Composition and Analysis,**14*(1), 3–13. 10.1006/jfca.2000.0960

[CR57] Nandi, R., Nedumaran, S., & Ravula, P. (2021). The interplay between food market access and farm household dietary diversity in low and middle income countries: A systematic review of literature. *Global Food Security,**28*, 100484. 10.1016/j.gfs.2020.100484

[CR58] Newton, P., Kinzer, A. T., Miller, D. C., Oldekop, J. A., & Agrawal, A. (2020). The number and spatial distribution of forest-proximate people globally. *One Earth,**3*(3), 363–370.

[CR59] Paumgarten, F., Locatelli, B., & Witkowski, E. T. F. (2018). Wild foods: Safety net or poverty trap? A South African case study. *Human Ecology,**46*(2), 183–195. 10.1007/s10745-018-9984-z

[CR60] Pawera, L., Khomsan, A., Zuhud, E. A. M., Hunter, D., Ickowitz, A., & Polesny, Z. (2020). Wild food plants and trends in their use: From knowledge and perceptions to drivers of change in West Sumatra, Indonesia. *Foods,**9*(9), 1240. 10.3390/foods9091240 32899857 10.3390/foods9091240 PMC7555794

[CR61] Pehlke, E. L., Letona, P., Ramirez-Zea, M., & Gittelsohn, J. (2016). Healthy *casetas* : A potential strategy to improve the food environment in low-income schools to reduce obesity in children in Guatemala City. *Ecology of Food and Nutrition,**55*(3), 324–338. 10.1080/03670244.2016.1161618 27065019 10.1080/03670244.2016.1161618 PMC4948585

[CR62] Phulkerd, S., Vandevijvere, S., Lawrence, M., Tangcharoensathien, V., & Sacks, G. (2017). Level of implementation of best practice policies for creating healthy food environments: Assessment by state and non-state actors in Thailand. *Public Health Nutrition,**20*(3), 381–390. 10.1017/S1368980016002391 27618938 10.1017/S1368980016002391 PMC10261628

[CR63] Pilling, D., & Bélanger, J. (2019). *The state of the world’s biodiversity for food and agriculture*. Rome: FAO Commission on Genetic Resources for Food and Agriculture.

[CR64] Pollom, T. R., Herlosky, K. N., Mabulla, I. A., & Crittenden, A. N. (2020). Changes in juvenile foraging behavior among the Hadza of Tanzania during early transition to a mixed-subsistence economy. *Human Nature,**31*(2), 123–140. 10.1007/s12110-020-09364-7 32458359 10.1007/s12110-020-09364-7

[CR65] Popkin, B. M., Corvalan, C., & Grummer-Strawn, L. M. (2020). Dynamics of the double burden of malnutrition and the changing nutrition reality. *The Lancet,**395*(10217), 65–74. 10.1016/S0140-6736(19)32497-3 10.1016/S0140-6736(19)32497-3 PMC7179702 31852602

[CR66] Powell, B., Kerr, R. B., Young, S. L., & Johns, T. (2017). The determinants of dietary diversity and nutrition: ethnonutrition knowledge of local people in the East Usambara Mountains. *Tanzania. Journal of Ethnobiology and Ethnomedicine,**13*, 23. 10.1186/s13002-017-0150-2 28449682 10.1186/s13002-017-0150-2 PMC5406938

[CR67] Powell, B., Maundu, P., Kuhnlein, H. V., & Johns, T. (2013). Wild Foods from Farm and Forest in the East Usambara Mountains, Tanzania. *Ecology of Food and Nutrition,**52*(6), 451–478. 10.1080/03670244.2013.768122 24083514 10.1080/03670244.2013.768122

[CR68] Powell, B., Thilsted, S. H., Ickowitz, A., Termote, C., Sunderland, T., & Herforth, A. (2015). Improving diets with wild and cultivated biodiversity from across the landscape. *Food Security,**7*(3), 535–554. 10.1007/s12571-015-0466-5

[CR69] Rasmussen, L. V., Coolsaet, B., Martin, A., Mertz, O., Pascual, U., Corbera, E., et al. (2018). Social-ecological outcomes of agricultural intensification. *Nature Sustainability,**1*(6), 275–282. 10.1038/s41893-018-0070-8

[CR70] Rasmussen, L. V., Fagan, M. E., Ickowitz, A., Wood, S. L. R., Kennedy, G., Powell, B., et al. (2019). Forest pattern, not just amount, influences dietary quality in five African countries. *Global Food Security*. 10.1016/j.gfs.2019.100331

[CR71] Rathi, N., Riddell, L., & Worsley, A. (2017). Food environment and policies in private schools in Kolkata, India. *Health Promotion International,**32*(2), 340–350. 10.1093/heapro/daw053 27402790 10.1093/heapro/daw053

[CR72] Reyes-García, V., Powell, B., Díaz-Reviriego, I., Fernández-Llamazares, Á., Gallois, S., & Gueze, M. (2019). Dietary transitions among three contemporary hunter-gatherers across the tropics. *Food Security,**11*(1), 109–122. 10.1007/s12571-018-0882-4

[CR73] Rowland, D., Ickowitz, A., Powell, B., Nasi, R., & Sunderland, T. (2017). Forest foods and healthy diets: Quantifying the contributions. *Environmental Conservation,**44*(2), 102–114. 10.1017/S0376892916000151

[CR74] Ruffo, C. K., Birnie, A., & Tengnäs, B. (2002). *Edible Wild Plants of Tanzania*. Regional Land Management Unit (RELMA).

[CR75] Saldana, J. (2021). *The Coding Manual for Qualitative Researchers*. Thousand Oaks, CA: SAGE Publications Limited.

[CR76] Schlegel, S. A., & Guthrie, H. A. (1973). Diet and the tiruray shift from swidden to plow farming. *Ecology of Food and Nutrition,**2*(3), 181–191. 10.1080/03670244.1973.9990335

[CR77] Schunko, C., Li, X., Klappoth, B., Lesi, F., Porcher, V., Porcuna-Ferrer, A., & Reyes-García, V. (2022). Local communities’ perceptions of wild edible plant and mushroom change: A systematic review. *Global Food Security,**32*, 100601. 10.1016/j.gfs.2021.100601

[CR78] Scoones, I., Melnyk, M., & Pretty, J. N. (1992). *The hidden harvest: Wild foods and agricultural systems, a literature review and annotated bibliography*. International Institute for Environment and Development, London, UK: The Sustainable Agriculture Programme.

[CR79] Sibhatu, K. T., Krishna, V. V., & Qaim, M. (2015). Production diversity and dietary diversity in smallholder farm households. *Proceedings of the National Academy of Sciences,**112*(34), 10657–10662. 10.1073/pnas.1510982112 10.1073/pnas.1510982112 PMC4553771 26261342

[CR80] Siegel, K. R., Ali, M. K., Srinivasiah, A., Nugent, R. A., & Narayan, K. M. V. (2014). Do we produce enough fruits and vegetables to meet global health need? *PLoS ONE,**9*(8). 10.1371/journal.pone.0104059 25099121 10.1371/journal.pone.0104059 PMC4123909

[CR81] Somnasang, P., & Moreno-Black, G. (2000). Knowing, gathering and eating: Knowledge and attitudes about wild food in an Isan village in Northeastern Thailand. *Journal of Ethnobiology,**20*(2), 197–216.

[CR82] Sunderland, T. C. H. (2011). Food security: Why is biodiversity important? *International Forestry Review,**13*(3), 265–274. 10.1505/146554811798293908

[CR83] Swinburn, B., Kraak, V., Rutter, H., Vandevijvere, S., Lobstein, T., Sacks, G., et al. (2015). Strengthening of accountability systems to create healthy food environments and reduce global obesity. *The Lancet,**385*(9986), 2534–2545. 10.1016/S0140-6736(14)61747-5 10.1016/S0140-6736(14)61747-5 25703108

[CR84] The World Bank. (2022). *Empowering Women - Expanding Access to Assets en Economic Opportunities. Tanzania Economic Update March 2022. (No. 17)*. Washington, DC: World Bank Publications, The World Bank Group.

[CR85] Tooze, J. A. (2020). *Estimating Usual Intakes from Dietary Surveys: Methodologic Challenges, Analysis Approaches, and Recommendations for Low- and Middle-Income Countries*. Washington, DC: Intake – Center for Dietary Assessment/FHI Solutions.

[CR86] Turner, C., Aggarwal, A., Walls, H., Herforth, A., Drewnowski, A., Coates, J., et al. (2018). Concepts and critical perspectives for food environment research: A global framework with implications for action in low- and middle-income countries. *Global Food Security,**18*, 93–101. 10.1016/j.gfs.2018.08.003

[CR87] Turner, C., Kadiyala, S., Anju, A., Coates, J., Drewnowski, A., Hawkes, C., et al. (2017). Food environment working group: Technical brief concepts and methods for food environment research in low and middle income countries. *Innovative Methods and Metrics for Agriculture and Nutrition Actions (IMMANA) programme. *London, UK: Agriculture, Nutrition and Health Academy Food Environments Working Group (ANH-FEWG).

[CR88] Turner, C., Kalamatianou, S., Drewnowski, A., Kulkarni, B., Kinra, S., & Kadiyala, S. (2020). Food environment research in lowand middle-income countries: a systematic scoping review. *Advances in Nutrition,**11*(2), 387–397. 10.1093/advances/nmz031 31079142 10.1093/advances/nmz031 PMC7442349

[CR89] United Republic of Tanzania. (1998). Tanzania National Forest Policy. Dar es Salaam, Tanzania.

[CR90] United Republic of Tanzania. (2002). The National Forest Act No. 14 of 2002. Dar es Salaam, Tanzania.

[CR91] United Republic of Tanzania. (2008). Participatory Forest Management. Dar es Salaam, Tanzania.

[CR92] United Republic of Tanzania. (2017). The Forest (Amendments) Regulations. G.N. No.255 of 2017. Dar es Salaam, Tanzania.

[CR93] United Republic of Tanzania. (2022). The WildLife Conservation Act. Government Notice No. 461

[CR94] Vinceti, B., Termote, C., Ickowitz, A., Powell, B., Kehlenbeck, K., & Hunter, D. (2013). The contribution of forests and trees to sustainable diets. *Sustainability,**5*(11), 4797–4824. 10.3390/su5114797

[CR95] van Vliet, N., Gonzalez, A., Nyumu, J., Muhindo, J., Paemelaere, E. A. D., Cerutti, P. O., & Nasi, R. (2022). Reducing wild meat sales and promoting local food security: Lessons learnt from a behavior change campaign in Yangambi, Democratic Republic of Congo. *Ethnobiology and Conservation*, *11*. 10.15451/EC2022-04-11.09-1-14

[CR96] Weinberger, K., & Swai, I. (2006). Consumption of traditional vegetables in central and Northeastern Tanzania. *Ecology of Food and Nutrition,**45*(2), 87–103. 10.1080/03670240500530626

[CR97] Wunder, S., Börner, J., Shively, G., & Wyman, M. (2014). Safety nets, gap filling and forests: A global-comparative perspective. *World Development,**64*, S29–S42. 10.1016/j.worlddev.2014.03.005

